# Eptinezumab for the prevention of chronic migraine: efficacy and safety through 24 weeks of treatment in the phase 3 PROMISE-2 (Prevention of migraine via intravenous ALD403 safety and efficacy–2) study

**DOI:** 10.1186/s10194-020-01186-3

**Published:** 2020-10-06

**Authors:** Stephen Silberstein, Merle Diamond, Nada A. Hindiyeh, David M. Biondi, Roger Cady, Joe Hirman, Brent Allan, Susan Pederson, Barbara Schaeffler, Jeff Smith

**Affiliations:** 1 Jefferson Headache Center, Department of Neurology, Thomas Jefferson University, Philadelphia, PA USA; 2 Diamond Headache Clinic, Chicago, IL USA; 3 Stanford University School of Medicine, Stanford, CA USA; 4 Cohen Veterans Bioscience, Cambridge, MA USA; 5 Lundbeck Seattle BioPharmaceuticals, Inc., Bothell, WA USA; 6 Pacific Northwest Statistical Consulting, Woodinville, WA USA

**Keywords:** Eptinezumab, Chronic migraine, Efficacy, Safety

## Abstract

**Background:**

PROMISE-2 was a phase 3, randomized, double-blind, placebo-controlled study that evaluated the efficacy and safety of repeat intravenous (IV) doses of the calcitonin gene-related peptide–targeted monoclonal antibody eptinezumab (ALD403) for migraine prevention in adults with chronic migraine. This report describes the results of PROMISE-2 through 24 weeks of treatment.

**Methods:**

Patients received up to two 30-min IV administrations of eptinezumab 100 mg, 300 mg, or placebo separated by 12 weeks. Patients recorded migraine and headache endpoints in a daily eDiary. Additional assessments, including patient-reported outcomes, were performed at regularly scheduled clinic visits throughout the 32-week study period (screening, day 0, and weeks 2, 4, 8, 12, 16, 20, 24, and 32).

**Results:**

A total of 1072 adults received treatment: eptinezumab 100 mg, *n* = 356; eptinezumab 300 mg, *n* = 350; placebo, *n* = 366. The reduction in mean monthly migraine days observed during the first dosing interval (100 mg, − 7.7 days; 300 mg, − 8.2 days; placebo, − 5.6 days) was further decreased after an additional dose (100 mg, − 8.2 days; 300 mg, − 8.8 days; placebo, − 6.2 days), with both doses of eptinezumab demonstrating consistently greater reductions from baseline compared to placebo. The ≥50% and ≥ 75% migraine responder rates (MRRs) increased after a second dose, with more eptinezumab-treated patients experiencing migraine response than placebo patients (≥50% MRRs weeks 13–24: 100 mg, 61.0%; 300 mg, 64.0%; placebo, 44.0%; and ≥ 75% MRRs weeks 13–24: 100 mg, 39.3%; 300 mg, 43.1%; placebo, 23.8%). The percentages of patients who improved on patient-reported outcomes, including the Headache Impact Test and Patient Global Impression of Change, increased following the second dose administration at week 12, and were greater with eptinezumab than with placebo at all time points. No new safety concerns were identified with the second dose regarding the incidence, nature, and severity of treatment-emergent adverse events.

**Conclusion:**

Eptinezumab 100 mg or 300 mg administered IV at day 0 and repeated at week 12 provided sustained migraine preventive benefit over a full 24 weeks and demonstrated an acceptable safety profile in patients with chronic migraine.

**Trial registration:**

ClinicalTrials.gov (Identifier: NCT02974153). Registered November 23, 2016.

## Background

Migraine, a highly prevalent neurological disorder, is characterized by recurrent episodes of moderate to severe headache associated with disruptions of neurological, gastrointestinal and sensory function often recurring over decades of life [1]. It is estimated that approximately 2.5% of persons with migraine transform from episodic to chronic migraine annually [2]. For those with chronic migraine, headaches are generally more intense, migraine-associated symptoms are more severe, and disease-related impact and disability are much greater than episodic migraine [3]. In addition, chronic migraine is associated with more comorbid diseases, such as anxiety, arthritis, chronic pain, and depression [4]. The high prevalence of migraine and migraine-related disability, especially with chronic migraine, is an important rationale for pursuing the development of effective therapeutics for migraine prevention.

Eptinezumab (Vyepti™, Lundbeck Seattle BioPharmaceuticals, Inc., Bothell, WA, USA) is a humanized monoclonal antibody that inhibits calcitonin gene-related peptide and was recently approved by the US Food & Drug Association for preventive treatment of migraine in adults. In the pivotal phase 3 PROMISE (PRevention Of Migraine via Intravenous ALD403 Safety and Efficacy) clinical trials of eptinezumab in patients with episodic migraine [5] and chronic migraine [6], eptinezumab 100 mg and 300 mg met the primary efficacy endpoints, with the majority of treatment-emergent adverse events (TEAEs) categorized as mild to moderate in severity.

The objective of this report is to describe the treatment effects of eptinezumab 100 mg and 300 mg for the prevention of chronic migraine over the full 24-week treatment period of the pivotal PROMISE-2 trial. PROMISE-2 was a phase 3, randomized, double-blind, placebo-controlled study designed to evaluate the efficacy, safety, and pharmacokinetics of repeat intravenous (IV) doses of the eptinezumab for migraine prevention in adults with chronic migraine. During the primary 12-week treatment period of PROMISE-2—the results of which have been published [6]—single doses of eptinezumab 100 mg and 300 mg were associated with significant reductions in the primary endpoint (mean monthly migraine days [MMDs]; *p* < 0.0001 vs placebo). Additionally, as a key secondary endpoint, the percentage of patients with a migraine reduced by over half on the day after the initial dose [6]. Both doses (100 mg and 300 mg) were associated with statistically significant and clinically meaningful migraine preventive effects over multiple efficacy measures and were well tolerated.

## Methods

### Study oversight

Study approval was provided by the independent ethics committee or institutional review board at each study site. The research was conducted in accordance with current Good Clinical Practices as referenced in the International Conference on Harmonization of Technical Requirements for Registration of Pharmaceuticals for Human Use guidelines, the principles of the Declaration of Helsinki, and local regulatory requirements. Before study initiation, each enrollee provided written informed consent prior to their participation. This study is registered on ***ClinicalTrials.gov*** under the following identifier: NCT02974153.

### Study design and procedures

**Fig. 1:**
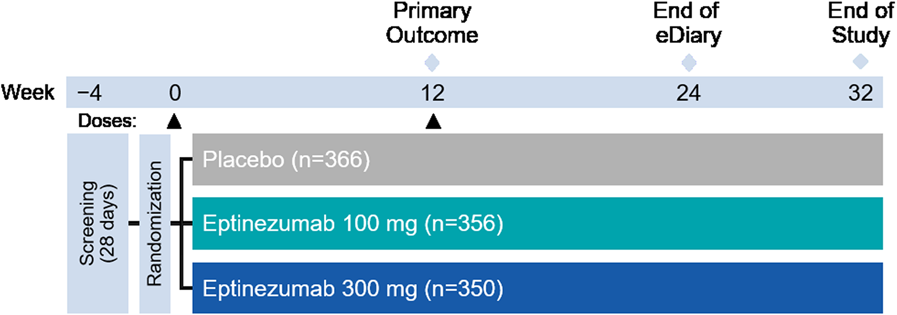
PROMISE-2 study design

Full study details have been published previously, including all inclusion and exclusion criteria [6]. Briefly, PROMISE-2 was conducted at 128 study sites in 13 countries between November 30, 2016, and April 20, 2018. Eligible patients were aged 18 to 65 years (inclusive), had a diagnosis of migraine at or before 50 years of age, had a ≥ 12-month history of chronic migraine prior to screening and, and experienced ≥15 to ≤26 headache days, including ≥ 8 migraine days, during the 28-day screening period (Fig. 1). Provided that dosages remained stable for ≥3 months prior to screening, preventive medication was allowed. Patients with medication-overuse headache (MOH) not associated with opioid analgesics or barbiturates were eligible.

Eligible patients were assigned randomly (1:1:1) to receive eptinezumab 100 mg, eptinezumab 300 mg, or placebo. Patient randomization was stratified by the number of migraine days experienced in the baseline period (≤17 days vs > 17 days) and by use of migraine preventive medication within the 3-month period before screening (use vs no use).

The entire study lasted 36 weeks and included scheduled visits at screening, day 0, and weeks 2, 4, 8, 12, 16, 20, 24, and 32. Patients received up to two IV doses of eptinezumab (100 mg or 300 mg) or placebo, on day 0 and at week 12 (administered over 30 min, plus another 15 min [per protocol, if required]). After completion of each dose, patients were monitored for at least two hours.

### Outcome measures

The daily eDiary was used through week 24 to obtain a daily report (irrespective of headache occurrence), and to capture headache events and migraine days, as well as acute use of medication. The start and end times for headaches were captured. For this study, a “migraine day” was defined as any day in which the patient had a migraine, established using the eDiary. A migraine was classified as lasting ≥4 h, or lasting 30 min to 4 h and believed by the patient to have been relieved by acute medication; having ≥2 of the following characteristics: unilateral location, pulsating quality, moderate or severe pain intensity, and/or aggravation by or causing avoidance of routine physical activity; and being accompanied by ≥1 of the following: nausea and/or vomiting or photophobia and phonophobia [1].

The primary efficacy endpoint (change from baseline in MMDs for weeks 1 through 12) has been reported [6]. In this analysis, mean change from baseline in MMDs was evaluated through the 24-week treatment period. Infusions of eptinezumab or placebo were provided at randomization and at the week 12 visit. Migraine efficacy results over each dosing interval (12-week period) were based upon the average number of MMDs occurring during each associated 4-week period. If the headache diary was completed for ≥21 days in a 4-week period, the observed frequency was normalized to 28 days. If the diary was completed for < 21 days in a 4-week period, the findings were a weighted function of the observed data for the current interval and the results for the previous interval, with the weight being proportional to the number of completed days.

Migraine responder rates were defined as the number of patients with a ≥ 50% and ≥ 75% reduction in MMDs from their baseline. These reductions were based on the reduction in the number of migraine days recorded in the eDiary during the baseline period compared with the average monthly number of migraine days recorded over weeks 1–12 and weeks 13–24. For the 100% responder endpoint, the 12-week 100% responder rate was the average of 100% responder rates for the three 4-week intervals. The 100% responder rate over weeks 13–24 was calculated using a similar methodology, utilizing data from weeks 13–16, 17–20, and 21–24.

Acute migraine medication days, defined as a day with any triptan or ergot use as recorded in the eDiary, were measured during the baseline period, and the mean change during each dosing interval (weeks 1–12 and 13–24) was calculated.

During the scheduled follow-up visits, patients self-reported different outcomes, including changes in their self-selected most bothersome symptom (MBS), and completed the 6-item Headache Impact Test (HIT-6 [7]; eptinezumab 300-mg group only), and the Patient Global Impression of Change (PGIC) [8] scale. At screening, patients described a MBS they associated with their chronic migraine; from this information, the investigator categorized the symptom into a predefined list, which included nausea, vomiting, sensitivity to light, sensitivity to sound, mental cloudiness, fatigue, pain with activity, and mood changes. The list also included an “Other, Specify” option for patient-identified symptoms that were outside of the predefined list. At weeks 4, 8, 12, 16, 20, 24, and 32, patients were asked to rate the overall change in that symptom since study initiation, using a scale identical in design to the PGIC [8] scale. For both scales, the seven possible responses were categorized as “very much improved”, “much improved”, “minimally improved”, “no change”, “minimally worse”, “much worse”, or “very much worse”. The HIT-6 v1.0 [7, 9]—which has been validated in patients with chronic migraine [10–12] —was administered at screening, day 0, and each study visit through week 32. Scores of ≥60 denote severe life impact, 56–59 indicate substantial life impact, 50–55 represent some life impact, and ≤ 49 demonstrates little or no life impact [13]; full details of the scoring system have been reported [6].

Safety was assessed throughout the study by monitoring adverse events (AEs) and treatment-emergent AEs (TEAEs), clinical laboratory tests, measuring vital signs, performing physical examinations and 12-lead electrocardiograms, completing the Columbia-Suicide Severity Rating Scale [14], and documenting concomitant medication use. TEAEs were classified according to the Medical Dictionary for Regulatory Activities (MedDRA), version 20.1.

Blood samples were collected at day 0 and weeks 2, 4, 8, 12, 24, and 32 to analyze pharmacokinetics and immunogenicity, which included monitoring for the development of anti-drug antibodies (ADAs) and assaying for neutralizing antibodies (NAbs).

### Statistical methods

The power calculations for PROMISE-2 have been reported [6]. All patients who received at least one dose of study medication were included in the efficacy and safety populations. For the safety analyses, results were summarized by study treatment. Results for patients who received two dose strengths for any reason were included in the highest-dose treatment group. For efficacy analyses, results were summarized for each randomization group.

A serial testing procedure was applied to account for multiplicity associated with testing multiple dose strengths and multiple endpoints. This procedure resulted in statistical significance for all endpoints and dose groups specified within the algorithm as reported in the primary manuscript [6]. Additional endpoints have been summarized here with descriptive statistics. Alpha-controlled endpoints have been previously summarized, and testing without alpha control has been conducted for select predefined endpoints. Least squares means from the ANCOVA model used to test the primary endpoint have been used to summarize the MMDs. All analyses were conducted with SAS software (SAS Institute, Cary, NC, USA) version 9.2 or higher.

## Results

### Patients

Patient disposition, as well as demographic and baseline clinical characteristics, have been presented previously [6]. A total of 1072 patients received treatment (eptinezumab 100 mg, *n* = 356; eptinezumab 300 mg, *n* = 350; placebo, *n* = 366) and were included in the efficacy and safety populations. Most patients (1020/1072 [95.1%]) in the eptinezumab treatment arms received both study infusions, including 340/356 (95.5%) in the 100 mg group and 338/350 (96.6%) in the 300 mg group.

Demographic and baseline clinical characteristics were balanced across treatment groups [6]. Briefly, the total patient population had a mean age of 40.5 years (standard deviation [SD], 11.2), with an average duration of migraine diagnosis of 18.1 years (SD, 11.8) and average duration of chronic migraine diagnosis of 11.8 years (SD, 11.2). Most patients were female (88.2%) and white (91.0%). During the 28-day screening period, patients experienced an average of 20.5 headache days (SD, 3.1), including 16.1 migraine days (SD, 4.6). A total of 1043 patients (97.3%) reported the use of ≥1 acute concomitant headache medication and 479 (44.7%) reported the use of ≥1 preventive concomitant headache medication; rates were well balanced across treatment groups. A total of 431 patients (40.2%) had a diagnosis of MOH per ICHD-3β (39%, 42%, and 40% of the eptinezumab 100 mg, 300 mg, and placebo groups, respectively).

### Migraine preventive efficacy

**Fig. 2:**
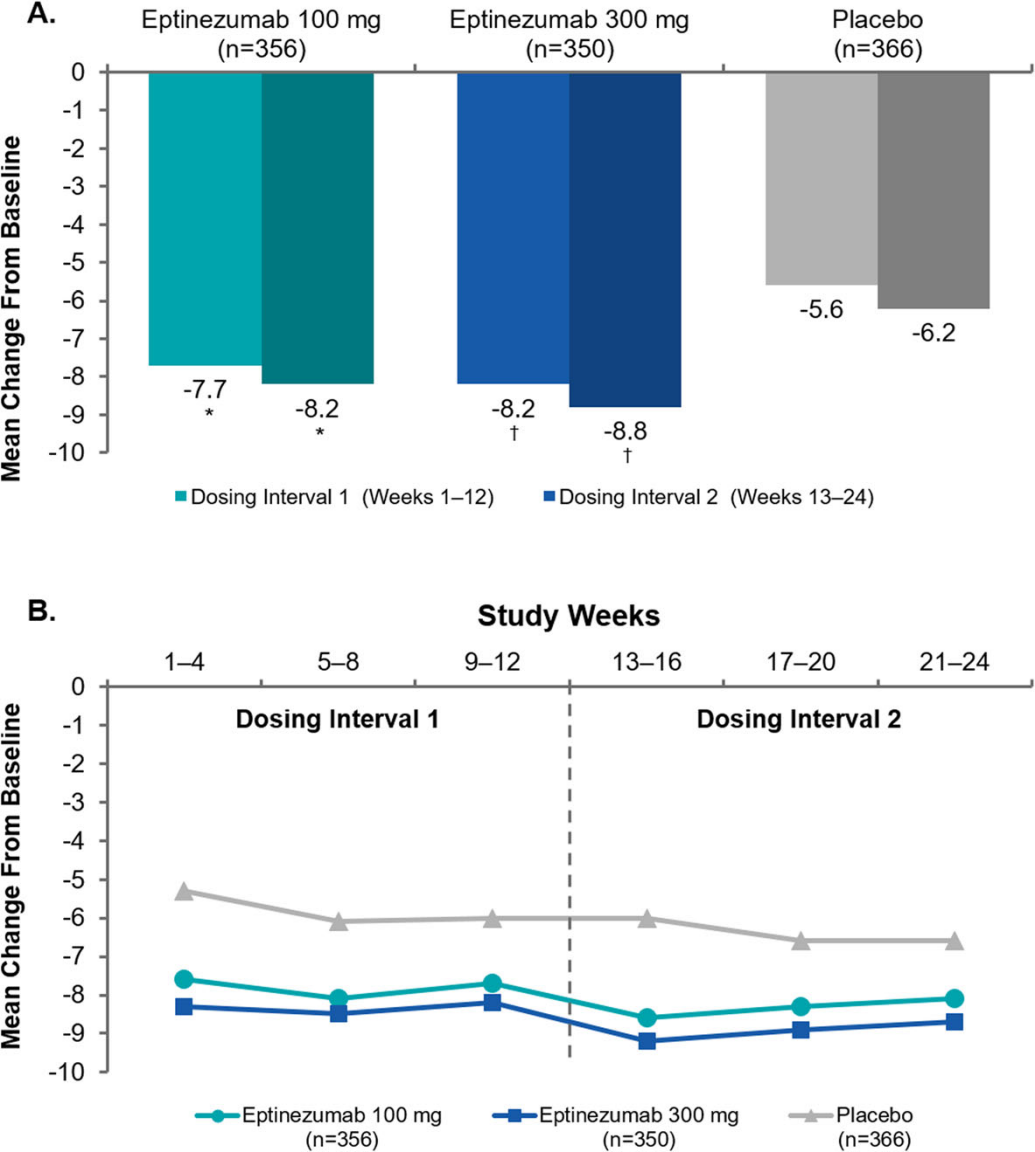
Reduction in mean monthly migraine days by (**a**) 12-week dosing interval and (**b**) 4-week interval (efficacy population). *P < 0.001 vs placebo. ^†^Nominal *P* < 0.001 vs placebo (analyses of monthly migraine days over weeks 13–24 were not formally tested per the predefined statistical hierarchy)

Efficacy outcomes for the two dosing intervals are summarized in Table 1. Eptinezumab treatment significantly reduced mean MMDs during the first dosing interval (weeks 1–12) compared with placebo, with incremental reductions observed during the second dosing interval (weeks 13–24) across all treatment arms (Fig. 2a). Reductions over 4-week intervals in MMDs were generally sustained across the 24-week treatment period, with eptinezumab 100 mg and 300 mg consistently resulting in greater reduction from baseline compared with placebo (Fig. 2b). Over weeks 1–24, the mean change from baseline in MMDs was − 7.9 for eptinezumab 100 mg, − 8.5 for 300 mg, and − 5.9 for placebo (difference from placebo: 100 mg, − 2.0 [95% CI: − 2.87, − 1.14]; 300 mg, − 2.6 [95% CI: − 3.49, − 1.76]). Reductions in mean headache days were consistent with the changes observed in MMDs during this time period.

**Table 1:** Efficacy outcomes (efficacy population)

	Weeks 1–12 (Dose 1)			Weeks 13–24 (Dose 2)		
	Eptinezumab 100 mg ***n*** = 356	Eptinezumab 300 mg ***n*** = 350	Placebo ***n*** = 366	Eptinezumab 100 mg ***n*** = 356	Eptinezumab 300 mg ***n*** = 350	Placebo ***n*** = 366
**Monthly migraine days**^**a**^						
Actual						
Mean	8.5	7.9	10.5	8.0	7.3	10.0
Change from baseline						
Mean	−7.7	−8.2	−5.6	−8.2	−8.8	−6.2
Difference from placebo	−2.03	−2.60		− 1.98	−2.65	
95% CI	(−2.88, − 1.18)	(− 3.45, − 1.74)		(− 2.94, − 1.01)	(− 3.62, − 1.68)	
**≥50% migraine responder rate**						
Patients, n (%)	205 (57.6)	215 (61.4)	144 (39.3)	217 (61.0)	224 (64.0)	161 (44.0)
Difference from placebo	18.2	22.1		17.0	20.0	
95% CI	(11.1, 25.4)	(14.9, 29.2)		(9.8, 24.1)	(12.9, 27.2)	
**≥75% migraine responder rate**						
Patients, n (%)	95 (26.7)	116 (33.1)	55 (15.0)	140 (39.3)	151 (43.1)	87 (23.8)
Difference from placebo	11.7	18.1		15.6	19.4	
95% CI	(5.8, 17.5)	(12.0, 24.3)		(8.9, 22.2)	(12.6, 26.2)	
**100% migraine responder rate**^**b**^						
Rate, %	10.8	15.1	5.1	17.8	20.8	9.3
Difference from placebo	5.8	10.1		8.5	11.5	
95% CI	(2.9, 8.6)	(6.7, 13.4)		(4.5, 12.5)	(7.2, 15.7)	
**Monthly headache days**						
Actual						
Mean	12.2 (6.68)	11.7 (6.96)	14.1 (6.38)	10.8 (7.42)	9.9 (7.44)	12.5 (7.25)
Change from baseline						
Mean	−8.2 (5.78)	−8.8 (6.10)	−6.4 (5.99)	−9.6 (6.62)	−10.6 (6.83)	−8.1 (6.90)
Difference from placebo	−1.7	−2.3		−1.5	−2.4	
95% CI	(−2.59, −0.87)	(−3.22, −1.44)		(−2.44, −0.47)	(−3.43, −1.42)	
**Acute migraine medication days**^**c**^						
Actual						
Mean	3.3 (4.84)	3.2 (4.71)	4.3 (5.67)	3.2 (4.94)	2.8 (4.49)	4.0 (5.59)
Change from baseline						
Mean	−3.3 (4.89)	− 3.5 (4.62)	−1.9 (4.18)	−3.4 (5.14)	−3.9 (4.96)	−2.2 (4.73)
Difference from placebo	−1.2	−1.4		−1.1	−1.7	
95% CI	(−1.66, −0.65)	(−1.88, −0.87)		(−1.86, −0.42)	(−2.44, −1.01)	

^a^The estimated mean, mean difference from placebo, and 95% confidence interval are from an analysis of covariance model, with treatment as a factor and the stratification variables; baseline migraine days and preventive medication use as independent variables

^b^Calculated as the average percentage of patients with 100% migraine response for any given 4-week study month during the respective dosing interval

^c^Defined as a day with any triptan or ergot use as recorded in the eDiary

*Abbreviations*: *CI* confidence interval, *MHD* monthly headache day, *MMD* monthly migraine day, *SD* standard deviation

**Fig. 3:**
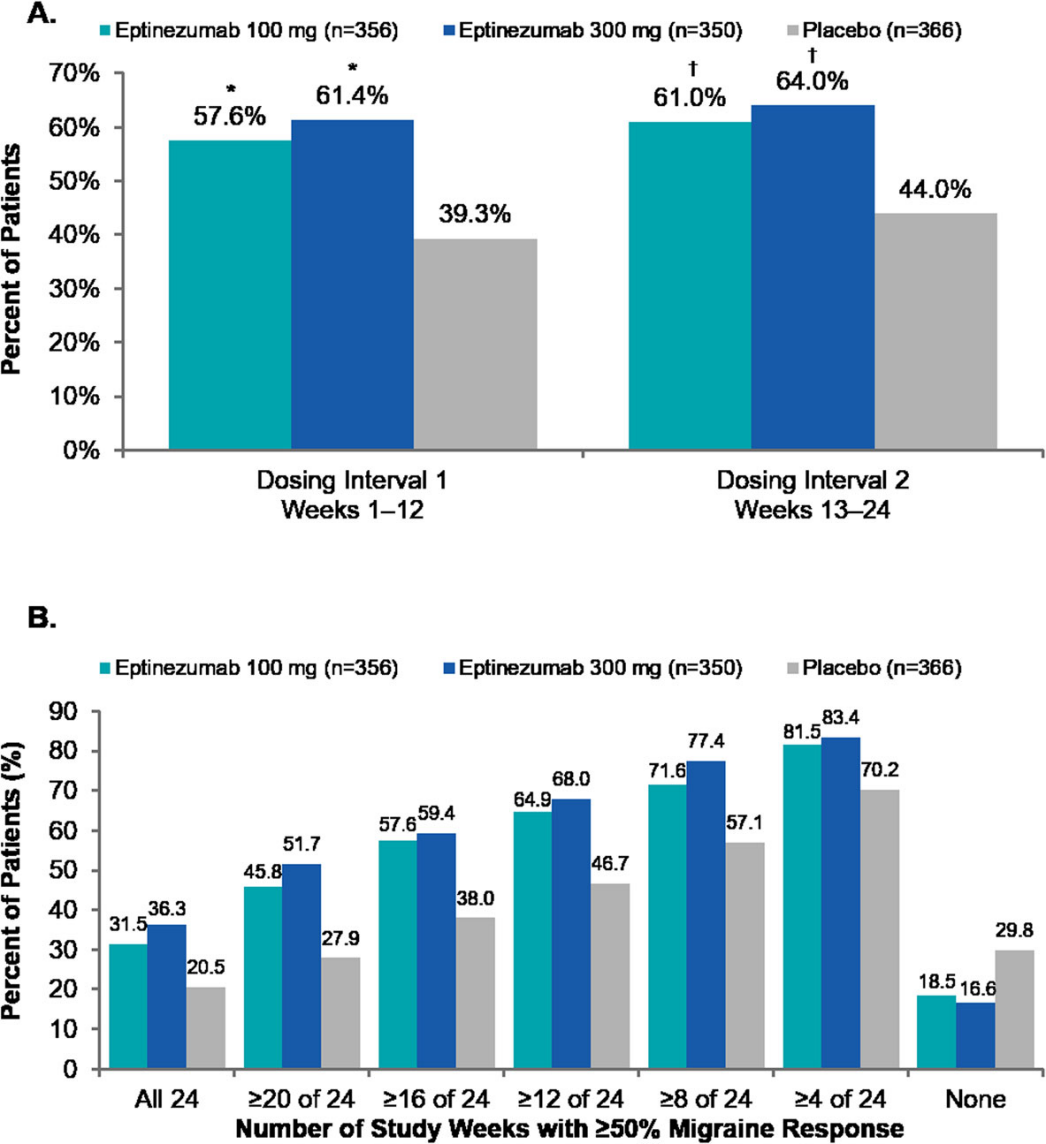
(**a**) Rates of ≥50% migraine response by 12-week dosing interval and (**b**) cumulative 4-week intervals with ≥50% migraine response (efficacy population). *P < 0.001 vs placebo. ^†^Nominal *P* < 0.001 vs placebo (analyses of migraine responder rates over weeks 13–24 were not formally tested per the predefined statistical hierarchy)

Eptinezumab 100 mg and 300 mg resulted in larger percentages of patients achieving ≥50% reduction in MMDs when compared with placebo across both dosing intervals (Fig. 3a). During the first dosing interval, the ≥50% migraine responder rates were significantly greater with eptinezumab (both doses), and this was sustained during the second dosing interval across all treatment groups. Post hoc analyses demonstrated that over 80% of patients reported ≥50% fewer migraine days in at least one 4-week interval, and approximately one-third of patients treated with eptinezumab (100 mg, 31.5%; 300 mg, 36.3%) were ≥ 50% migraine responders for the entire treatment period (i.e., in each 4-week interval from Weeks 1 to 24) compared with 20.5% of patients who received placebo (Fig. 3b). The odds ratio (versus placebo) of cumulative months with a ≥ 50% migraine response was 1.974 (95% CI: 1.522, 2.561) for eptinezumab 100 mg and 2.389 (95% CI: 1.836, 3.110) for eptinezumab 300 mg.

**Fig. 4:**
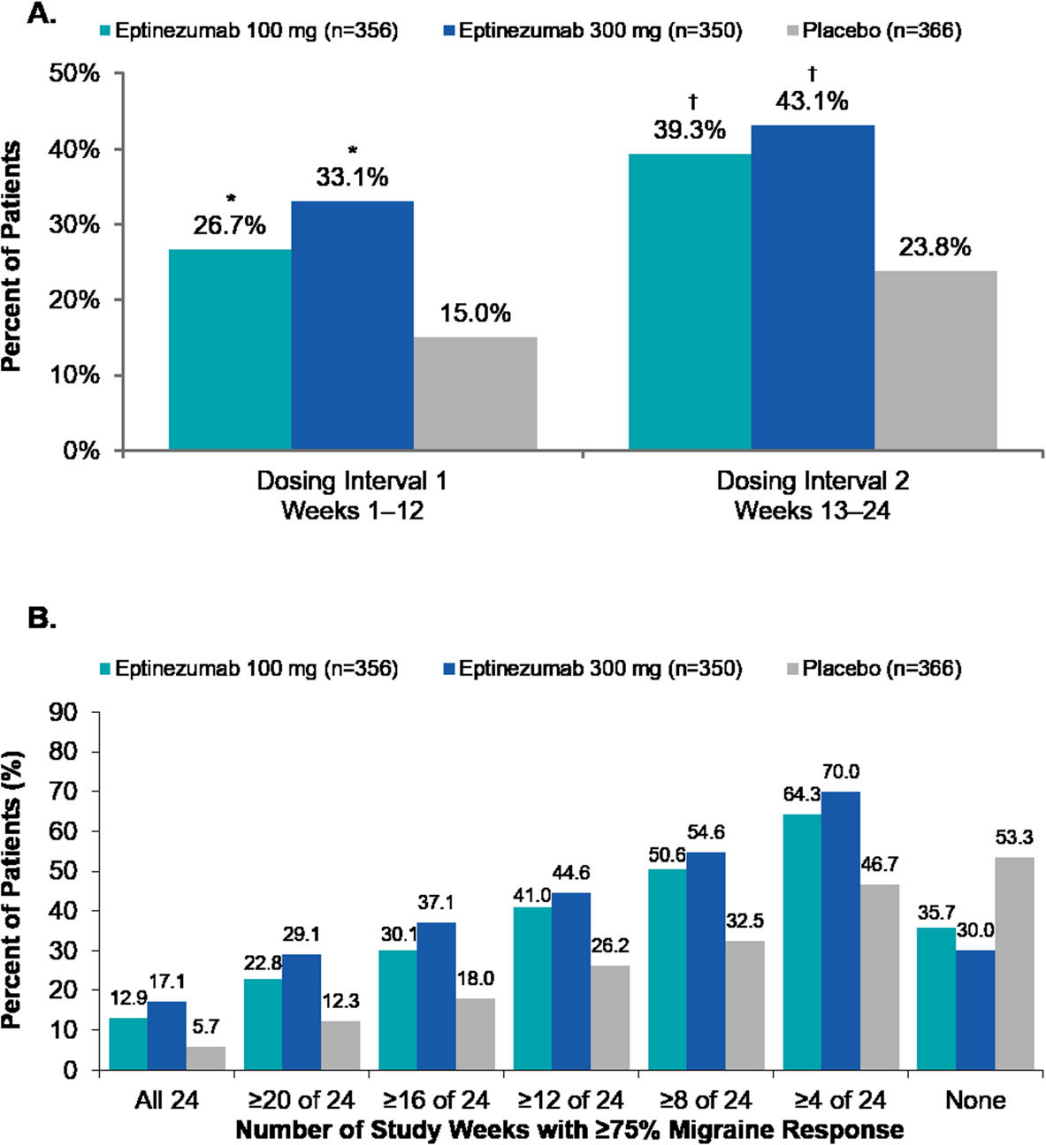
(**a**) Rates of ≥75% migraine response by 12-week dosing interval and (**b**) cumulative 4-week intervals with ≥75% migraine response (efficacy population). *P ≤ 0.001 vs placebo

The percentage of patients with a ≥ 75% reduction in MMDs across the 2 dosing intervals was consistently higher in the eptinezumab (100 mg and 300 mg) treatment groups compared with placebo (Fig. 4a). During the first dosing interval, the ≥75% migraine responder rates were significantly greater than placebo, with similar rate increases during the second dosing interval in all treatment groups. Post hoc analyses demonstrated that 64–70% of patients achieved at least one 4-week interval with a ≥ 75% reduction in monthly migraine frequency, and approximately 13% and 17% of patients treated with eptinezumab 100 mg and 300 mg, respectively, were ≥ 75% migraine responders for all 4-week intervals through Week 24 (compared with 46.7% and 5.7% of patients who received placebo, respectively) (Fig. 4b). The odds ratio (versus placebo) of the cumulative number of weeks with a ≥ 75% migraine response was 2.057 (95% CI: 1.572, 2.691) for eptinezumab 100 mg and 2.637 (95% CI: 2.012, 3.456) for 300 mg.

**Fig. 5:**
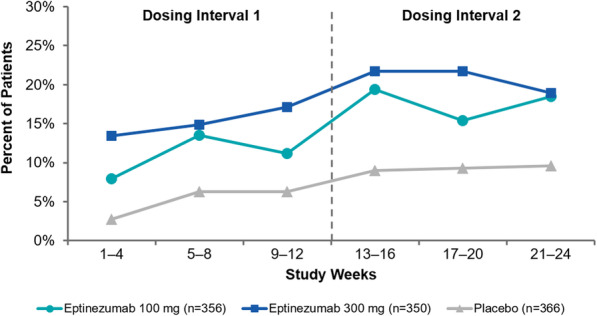
Rates of 100% migraine response by 4-week interval (efficacy population). ^†^Nominal *P* < 0.001 vs placebo (analyses of migraine responder rates over weeks 13–24 were not formally tested per the predefined statistical hierarchy)

The percentage of patients with 100% monthly migraine response ranged from 7.9% to 19.4% for eptinezumab 100 mg and 13.4% to 21.7% for eptinezumab 300 mg compared with 2.7% to 9.6% for placebo. During each 4-week interval of the study, the percentage of patients with a 100% reduction in migraine days (i.e. patients with no migraines for a 4-week study month) was higher in the eptinezumab groups than in the placebo group (Fig. 5). The average of the 4-week 100% migraine responder rate over weeks 1–12 was 10.8% for eptinezumab 100 mg and 15.1% for eptinezumab 300 mg, compared with 5.1% for the patients who received placebo. During the second dosing interval, the percentage of patients experiencing ≥4 weeks with 100% migraine response increased to 17.8% with eptinezumab 100 mg, 20.8% with eptinezumab 300 mg, and 9.3% with placebo.

In the 12-week analysis, both doses of eptinezumab were associated with reduced acute migraine medication days from baseline to week 12, which were greater than the reduction associated with placebo—the difference from placebo was − 1.2 days for the 100-mg dose and − 1.4 days for the 300-mg dose. As shown in Table 1, during the second dosing interval (weeks 13–24) these differences in the reduction of acute migraine medication days were maintained or increased (difference from placebo was − 1.1 days for the 100-mg dose and − 1.7 days for the 300-mg dose).

### Impact of treatment on patient-reported outcomes

The effects of eptinezumab versus placebo on patient-reported outcome measures are summarized in Supplementary Table 1. At baseline, based on HIT-6 total scores, 89.6%, 88.6%, and 87.4% of patients in the eptinezumab 100 mg, 300 mg, and placebo treatment arms, respectively, had severe headache-related life impact (i.e., a score of ≥60). The percentage of patients with severe life impact dropped to 51.4% with eptinezumab 100 mg and 42.9% with 300 mg compared with 60.1% in the placebo group at week 12. The percentage of patients with severe life impact further decreased during the second dosing interval, comprising 43.5%, 39.7%, and 55.3% of patients in the eptinezumab 100 mg, 300 mg, and placebo groups, respectively, by week 24. Correspondingly, the percentages of patients with little to no headache-related impact (HIT-6 total score ≤ 49) increased from < 1% at baseline to 14.0% and 16.4% at week 12 and week 24, respectively, for eptinezumab 100 mg and from 1.1% to 16.6% and 23.6%, respectively, for 300 mg. For placebo, the corresponding percentages were 1.4% at baseline, 9.6% at week 12, and 11.8% at week 24.

At week 12, more than half of eptinezumab-treated patients reported that their MBS was “much improved” or “very much improved”: 53.5% with eptinezumab 100 mg, 61.2% with eptinezumab 300 mg; this compared with 34.1% with placebo. At week 24, results were similar: eptinezumab 100 mg, 56.8%; 300 mg, 62.4%; placebo, 39.3%. On the PGIC, 52.3% of patients treated with eptinezumab 100 mg and 63.8% treated with eptinezumab 300 mg indicated “much improved” or “very much improved”, compared with 37.9% of placebo patients. PGIC improvements at week 24 increased, similarly to patient-identified MBS: eptinezumab 100 mg, 59.3%; 300 mg, 63.6%; placebo, 40.9%.

### Adverse events

Full safety data across 24-weeks of treatment have been presented previously [6]. Overall, 508 patients (47.4%) experienced ≥1 TEAE during the study, and rates across the treatment groups were similar. The percentages of patients experiencing any TEAE in the second dosing interval were lower than in the first dosing interval across all treatment groups (Table 2). TEAEs occurring in ≥2% of patients in any treatment arm in either dosing interval included nasopharyngitis, upper respiratory tract infection, migraine, and nausea. During both dosing intervals, TEAEs coded to hypersensitivity occurred in 3 patients (0.9%) treated with eptinezumab 300 mg.

**Table 2:** Treatment-emergent adverse events reported in ≥2% of patients in any treatment group by dosing interval (safety population)

Dosing Interval	Eptinezumab 100 mg			Eptinezumab 300 mg			Placebo		
	Total ***n*** = 356	Dose 1 ***n*** = 356	Dose 2 ***n*** = 348	Total ***n*** = 350	Dose 1 ***n*** = 350	Dose 2 ***n*** = 344	Total ***n*** = 366	Dose 1 ***n*** = 366	Dose 2 ***n*** = 356
Any event, n (%)	155 (43.5)	106 (29.8)	64 (18.4)	182 (52.0)	120 (34.3)	99 (28.8)	171 (46.7)	120 (32.8)	79 (22.2)
Nasopharyngitis	19 (5.3)	*12 (3.4)*	6 (1.7)	33 (9.4)	*19 (5.4)*	*7 (2.0)*	22 (6.0)	*11 (3.0)*	*11 (3.1)*
URTI	15 (4.2)	5 (1.4)	6 (1.7)	19 (5.4)	*8 (2.3)*	*9 (2.6)*	20 (5.5)	*13 (3.6)*	4 (1.1)
Migraine	6 (1.7)	1 (0.3)	4 (1.1)	8 (2.3)	2 (0.6)	4 (1.2)	16 (4.4)	6 (1.6)	*7 (2.0)*
Nausea	6 (1.7)	4 (1.1)	2 (0.6)	12 (3.4)	*10 (2.9)*	4 (1.2)	7 (1.9)	4 (1.1)	3 (0.8)

Dosing intervals were not mutually exclusive, meaning that a patient could be counted in both dosing intervals. Italics indicates ≥2% of patients for individual events. *URTI* upper respiratory tract infection

### Immunogenicity

The ADA response profile was similar in patients receiving either the 100 mg or 300 mg dose of eptinezumab. Maximal ADA incidence occurred at week 24, corresponding to 17.2% and 17.0% of treated patients in the 100 mg and 300 mg groups, respectively. ADA titers were low across all groups, with no trend of increasing titer related to dose. The ADA-positive incidence declined following the 24-week time point, and by week 32 the ADA-positive responses decreased to 9.0% (29/321) in the 300 mg group and 11.4% (37/325) in the 100 mg group. The overall NAb-positive incidence was 6.4% (45/706) of all eptinezumab-treated patients, with 26 patients treated with 100 mg and 19 patients treated with 300 mg having ADA-positive results with neutralizing potential. As indicated in the primary report [6], the development of ADA, including NAb, had no impact on the safety or efficacy outcomes with eptinezumab treatment.

Patients (66/646) who tested positive for anti-eptinezumab antibodies at the end-of-study visit (week 32) continued immunogenicity testing at approximately 12-week intervals for up to 24 weeks (i.e. follow-up visits) to evaluate persistence of the ADA response. At the 12-week follow-up visit, 60 ADA-positive patients were available for analysis, of whom 15 remained positive; of these, 5 had neutralizing antibodies. At the 24-week follow-up visit, 13 ADA patients were available; of these, 7 remained ADA-positive. Only 1 of these patients had neutralizing antibody. The number of ADA-positive subjects at the 12-week follow-up analysis were similar across the eptinezumab 100 mg and 300 mg dose groups, and the ADA responses at both the 12- and 24-week follow-up visits showed low titers (≤450) with a steady decline over time.

## Discussion

In this analysis of both 12-week treatment periods of the PROMISE-2 study, the magnitude of efficacy that eptinezumab achieved during the first dosing interval was sustained through 24 weeks, including both migraine-preventive effects and improvements on patient-reported outcomes. In addition, the temporal analysis of safety did not identify any new safety concerns with an additional dose.

Prior studies have shown that increased migraine frequency is associated with higher levels of medication usage, greater direct and indirect healthcare costs, and lower work productivity [15, 16]. The observed reductions in migraine frequency in the PROMISE-2 study could translate into decreased morbidity, reduced healthcare resource utilization, and lower rates of absenteeism and presenteeism. From an average baseline of 16 monthly migraine days, eptinezumab-treated patients had approximately 8 fewer migraine days each month on average relative to baseline and approximately 2 days fewer relative to placebo. The placebo effect observed in PROMISE-2 may be due to the route of administration, frequency of on-site visits, patient expectations and beliefs, or other contextual factors [17–20]. Despite the placebo response, eptinezumab demonstrated statistically and nominally different improvements in migraine frequency across 24 weeks of treatment.

The results for the reduction in monthly migraine days reflects the 61%–64% of eptinezumab-treated patients who experienced a ≥ 50% reduction in migraine compared with the 44% of placebo patients. Notably, approximately one-third of eptinezumab-treated patients reported a ≥ 50% reduction in migraine frequency over the entire 24-week treatment period, which could suggest the potential for remission from chronic migraine depending a patient’s baseline MMDs. For those who experienced a ≥ 75% reduction in migraine frequency, the reduction from the baseline average equates to 4 MMDs or less. A consistent ≥75% migraine response (defined as 24 weeks of ≥75% reduction) was achieved by 13%–17% of eptinezumab-treated patients compared with < 6% of placebo patients. Lastly, the percentage of patients treated with eptinezumab who experienced a 100% reduction in migraine increased as the study progressed; placebo rates increased to a lesser degree.

Early reports suggested that eptinezumab improves patient functioning, as assessed by patient-reported outcome measures [21, 22]. In a phase 2b dose-ranging study in patients with chronic migraine, mean HIT-6 scores improved by 6.9 in the eptinezumab 100 mg group and 10.0 in the 300 mg group versus 5.8 for the placebo group [22]. In the current study, the percentage of patients in the eptinezumab groups reporting severe headache-related life impact (based on HIT-6 total scores) was decreased by approximately half at the week 12 and 24 visits, compared with approximately 40% reduction with placebo patients. At the other end of the HIT-6 spectrum, the percentage of patients reporting little to no headache-related life impact increased more in the eptinezumab groups than in the placebo group at both week 12 and 24. Similar patterns were observed in the patient-identified MBS and PGIC measure, with ~ 60% of eptinezumab-treated patients reporting “much improved” or “very much improved” at week 24 on each patient-reported outcome vs ~ 40% in the in the placebo group. While the PGIC measures patient-perceived improvements in disease status, patient-identified MBS may be an additional determinant of patient satisfaction that is not captured in other common patient-reported outcome measure or not associated with reductions in MMDs. These findings show that reductions in migraine frequency translate into meaningful improvements in patients’ daily lives, allowing them to more fully participate in daily activities such as those associated with work, school, and/or social pursuits. Migraine has an established negative impact on daily life and functioning, with burdens placed on patients, as well as on their families and caregivers [23–25]. Improvements in functioning are, therefore, a key indicator of meaningful day-to-day treatment benefit for patients.

We have previously reported that eptinezumab significantly reduced MMDs over weeks 1–12 after the first IV administration, and this could be observed from as early as day 1 following treatment [6]. Early onset and sustained response during the PROMISE-2 study may be a result of the pharmacokinetic properties of eptinezumab and its route of administration. Maximum plasma concentrations are achieved immediately upon completion of delivery of the IV administration and persist throughout the dosing interval with a terminal elimination half-life of 27 days [26, 27].

Eptinezumab was well tolerated throughout the 24 weeks of treatment, with no change in the safety profile identified with a second dose. Due to the chronic nature of chronic migraine, it would be expected that patients eligible for preventive treatment would be repeatedly dosed; therefore, it is important to understand any potential cumulative effects of repeated exposure. Overall, rates of TEAEs had no clear dose-response trend and were generally similar to placebo across both dosing intervals, demonstrating the safety and tolerability of repeated doses of eptinezumab in patients with migraine. Of note is the lack of a safety signal with regard to constipation and hypertension, two side effects which have been reported in post-marketing surveillance studies of other approved monoclonal antibodies targeting calcitonin gene-related peptide [28]. Adverse events are often the reason for preventive treatment discontinuation [29]; therefore, continued good tolerability is important to maintain persistence with therapy.

In previous eptinezumab studies the ADA response was maximal at week 24 [5, 22] and declined thereafter. The transient nature of the eptinezumab-associated ADA response was demonstrated again in the current study, with ADA-positive incidence declining after week 24 and through the end of the study (week 32), with no observed effect on the efficacy or safety of eptinezumab treatment. At the 12-week follow-up analysis, there were no notable differences in the number of ADA-positive patients between eptinezumab 100 mg and 300 mg dose groups, indicating that persistence was not dose-related.

### Study limitations

The current study was of sufficient duration to demonstrate the efficacy of eptinezumab in patients with chronic migraine, but the safety and durability of response over prolonged periods of use remain to be fully evaluated. The results of the open-label PREVAIL safety study (ClinicalTrials.gov: NCT02985398), which examined the safety and tolerability of long-term treatment with eptinezumab, will be reported separately. Generalizability of the results of this study is limited by the exclusion of certain patient populations**.**

## Conclusions

In adult patients with chronic migraine, eptinezumab 100 mg and 300 mg administered by 30-min IV on day 0 and at week 12 provided sustained migraine preventive benefit over 24 weeks of treatment. In addition to the majority (~ 60%) of eptinezumab-treated patients experiencing a ≥ 50% reduction in migraine frequency (and ~ 40% experiencing a ≥ 75% reduction), treatment with eptinezumab was associated with meaningful improvements in patient-reported outcomes measuring headache-related life impact, patient perception of disease, and patient-identified MBS. A second dose of eptinezumab did not result in any new safety signals, including immunogenetic reactions. In conclusion, this report provides further evidence that eptinezumab IV can provide early and sustained migraine prevention and improvements in patient functioning, with low risk of adverse events.

## Supplementary information

**Additional file 1: Supplementary Table 1.** Summary of patient-reported outcomes measures (PROs) by visit and treatment.

## Data Availability

The data reported in this manuscript are part of an ongoing, global sponsor-led clinical development and registration program. Deidentified participant data are not available for legal and ethical reasons.

## Abbreviations

ADA: Anti-drug antibody
AE: Adverse event
ANCOVA: Analysis of covariance
C-SSRS: Columbia-Suicide Severity Rating Scale
CMH: Cochran-Mantel-Haenszel
ECG: Electrocardiogram
HIT-6: 6-item Headache Impact Test
IV: Intravenous
MBS: Most bothersome symptom
MMD: Monthly migraine day
NAb: Neutralizing antibody
PGIC: Patient Global Impression of Change
TEAE: Treatment-emergent adverse event
