# Supplementary material for: Eptinezumab for the prevention of chronic migraine: efficacy and safety through 24 weeks of treatment in the phase 3 PROMISE-2 (Prevention of migraine via intravenous ALD403 safety and efficacy–2) study
Source: J Headache Pain. 2020 Oct 6;21(1):120. doi: 10.1186/s10194-020-01186-3 (PMC7539382; doi:10.1186/s10194-020-01186-3)
Supplement: Supplementary file 1 — Additional file 1: Supplementary Table 1. Summary of patient-reported outcomes measures (PROs) by visit and treatment. [file 10194_2020_1186_MOESM1_ESM.docx]

**Supplementary Table 1** Summary of patient-reported outcomes measures (PROs) by visit and treatment

|  |  | **Eptinezumab 100 mg  *n* = 356** | **Eptinezumab 300 mg *n* = 350** | **Placebo  *n* = 366** |
| --- | --- | --- | --- | --- |
| **HIT-6 Life Impact, *n* (%)^a^** | | | | |
| Baseline | Severe Substantial Some Little to none | 319 (89.6) 27 (7.6) 8 (2.2) 2 (<1) | 310 (88.6) 28 (8.0) 8 (2.3) 4 (1.1) | 320 (87.4) 26 (7.1) 15 (4.1) 5 (1.4) |
| Weeks 9–12 | Severe Substantial Moderate Little to none | 183 (51.4) 66 (18.5) 57 (16.0) 50 (14.0) | 150 (42.9) 59 (16.9) 83 (23.7) 58 (16.6) | 220 (60.1) 58 (15.8) 53 (14.5) 35 (9.6) |
| Weeks 21–24 | Severe Substantial Moderate Little to none | 143 (43.5) 67 (20.4) 65 (19.8) 54 (16.4) | 131 (39.7) 51 (15.5) 70 (21.2) 78 (23.6) | 183 (55.3) 44 (13.3) 65 (19.6) 39 (11.8) |
| **PGIC, *n* (%)** |  |  |  |  |
| Week 12 | *n* Very much improved Much improved Minimally improved No change Worse^b^ | 344 54 (15.7) 126 (36.6) 82 (23.8) 69 (20.1) 13 (3.8) | 337 73 (21.7) 142 (42.1) 68 (20.2) 51 (15.1) 3 (<1) | 343 38 (11.1) 92 (26.8) 85 (24.8) 111 (32.4) 17 (5.0) |
| Week 24 | *n* Very much improved Much improved Minimally improved No change Worse^b^ | 329 53 (16.1) 142 (43.2) 71 (21.6) 49 (14.9) 14 (4.3) | 330 93 (28.2) 117 (35.5) 56 (17.0) 54 (16.4) 10 (3.0) | 331 51 (15.4) 84 (25.4) 72 (21.8) 99 (29.9) 25 (7.6) |
| **Patient-Identified MBS, *n* (%)** | | | | |
| Week 12 | *n* Very much improved Much improved Minimally improved No change Worse^b^ | 344 54 (15.7) 130 (37.8) 84 (24.4) 61 (17.7) 15 (4.4) | 338 79 (23.4) 128 (37.9) 75 (22.2) 52 (15.4) 4 (1.2) | 343 41 (12.0) 76 (22.2) 111 (32.4) 101 (29.4) 14 (4.1) |
| Week 24 | *n* Very much improved Much improved Minimally improved No change Worse^b^ | 329 64 (19.5) 123 (37.4) 71 (21.6) 56 (17.0) 15 (4.6) | 330 92 (27.9) 114 (34.5) 61 (18.5) 52 (15.8) 11 (3.3) | 331 53 (16.0) 77 (23.3) 76 (23.0) 101 (30.5) 24 (7.3) |

^a^Score ranges are associated with the following life impact: ≥60, severe; 56–59, substantial; 50–55, moderate; ≤49, little to none.

^b^Worse includes “minimally worse”, “much worse”, and “very much worse”.

Abbreviations: CI: confidence interval; HIT-6: Headache Impact Test-6; MBS: most bothersome symptom; PCIC: Patient Global Impression of Change.
